# Acceptability and Feasibility of a Sedentary Behavior Reduction Program during Pregnancy: A Semi-Experimental Study

**DOI:** 10.3390/healthcare8040439

**Published:** 2020-10-29

**Authors:** Maiko Kawajiri, Yasuka Nakamura, Mikako Yoshida, Yoko Takeishi, Ai Masaki, Yuki Iwasaki, Satomi Sato, Yuri Kodera, Kazumi Chiba, Toyoko Yoshizawa

**Affiliations:** 1 Department of Women’s Health Nursing & Midwifery, Tohoku University Graduate School of Medicine, Sendai, Miyagi 9808575, Japan; nakamurayasuka@nursing.med.tohoku.ac.jp (Y.N.); mikako.yoshida.e2@tohoku.ac.jp (M.Y.); yoko.takeishi@med.tohoku.ac.jp (Y.T.); toyoko@nursing.med.tohoku.ac.jp (T.Y.); 2 Department of Nursing, Tohoku University Hospital, Sendai, Miyagi 9808575, Japan; ai.masaki.d5@tohoku.ac.jp (A.M.); liberteester0205@gmail.com (Y.I.); satomi.sato.d8@tohoku.ac.jp (S.S.); yuri.kodera.c7@tohoku.ac.jp (Y.K.); kazumi.chiba.e2@tohoku.ac.jp (K.C.)

**Keywords:** accelerometer, behavioral change, mobile health, nursing intervention, pregnancy, sitting time

## Abstract

Physical activity (PA) during pregnancy is associated with maternal benefits. Therefore, innovative strategies that promote PA are needed. This study investigated the acceptability and feasibility of a sedentary behavior (SB) reduction program during pregnancy. The study employed a semi-experimental research design using historical control subjects. The intervention group program consisted of individual face-to-face guidance, automatic alerts during SB from wearable devices, and self-monitoring of SB patterns, from 20 gestation weeks to delivery. PA and SB, assessed using a wearable device, were compared with those of the control group at 24–27 (T1) and 32–35 (T2) weeks of gestation. In 56 women, the mean wearing time was 90.2 days in the intervention phase. The response rate to automatic SB alerts was 55.5% at T1 and 63.0% at T2. Self-monitoring more than twice or thrice a week was 77.8% at T1 and 59.3% at T2. There was no significant difference in the cumulative SB time at T2 between the two groups (F = 2.31, *p* = 0.132). This program appears to be acceptable and feasible for pregnant women; however, SB reduction effect of the intervention remains unclear. Improvements to increase the response rate to automatic SB alerts and the frequency of self-monitoring are needed.

## 1. Introduction

Maintaining regular physical activity (PA) during pregnancy is associated with physical and psychological benefits, including appropriate weight gain, and reduced risk of gestational diabetes and postpartum depression [1,2]. Although the American College of Obstetricians and Gynecologists recommends that low-risk pregnant women engage in 30 min of moderate PA at least 5 days per week [3,4], most women are unable to meet this recommendation [5,6,7]. Thus, national public health strategies are needed to encourage pregnant women to increase their PA.

Most interventions designed to improve PA among pregnant women have focused on increasing moderate PA with exercise programs. However, these programs (a 1–2 times per week group exercise with moderate PA) have been shown to have high dropout rates and lower adherence to protocols [8,9,10,11,12]. In recent years, some interventions have focused on increasing moderate PA in activities of daily living [13,14] but have not been successful compared to previous exercise interventions. Lack of time [15,16,17], physical limitations, fatigue [17], and concerns about preterm birth [18] are major barriers to participation and improvement in PA through exercise programs involving lifestyle activities.

In recent years, sedentary behavior (SB), defined as any waking behavior characterized by an energy expenditure less than or equal to 1.5 metabolic equivalents, has been a focal point in promoting PA in the general population. The World Health Organization’s Global Action Plan on PA 2018–2030 suggests that reducing SB through the promotion of incidental PA (e.g., standing, climbing stairs, and short walks) can encourage individuals to increase their PA to achieve the recommended levels for optimal health. Interventions focusing on reducing SB have been reported to significantly increase energy expenditure [19] and have high adherence in populations in whom it is traditionally difficult to promote exercise, such as obese adults or the elderly [20,21]. Therefore, these interventions, which are low-intensity, safe, and can be performed in daily life, are more likely to be effective for pregnant women.

Interventions designed to reduce SB during pregnancy have not been reported to date. It is necessary to evaluate the feasibility of the intervention and if the intervention results in increased PA in pregnant women, before investigating its effect on reducing pregnancy complications is warranted. The aim of this study was to evaluate the acceptability and feasibility of an SB reduction program during pregnancy.

## 2. Materials and Methods

A semi-experimental study using historical control subjects was conducted between July 2018 and June 2019 in an urban area in the Tohoku region of Japan. The intervention group participated in an SB reduction program for pregnant women from 20 weeks of gestation to delivery. For the control group, data from a previous study describing the changes in PA and SB among pregnant women [22] were used. PA and SB were compared at 24–27 (T1) and 32–35 (T2) weeks of gestation, respectively, in the control and intervention groups. All subjects gave their informed consent for inclusion before they participated in the study. The study was conducted in accordance with the Declaration of Helsinki, and the protocol was approved by the Ethics Committee of Tohoku University Graduate School of Medicine (2016-1-086, 2018-1-181).

Healthy pregnant women expecting their first infant were recruited at obstetric clinics and hospitals as part of the intervention group. To recruit participants, posters with details of the research study were placed on bulletin boards in healthcare facilities and flyers were distributed to pregnant women enrolled in prenatal health classes. Pregnant women who were interested in participating in the study emailed the researcher or spoke to hospital healthcare staff. The inclusion criteria were (1) less than 19 weeks of gestation, (2) singleton pregnancy, (3) ability to participate in the study until delivery, (4) being at least 20 years of age, and (5) having the ability to read and write Japanese. The exclusion criteria were (1) an obstetric or medical restriction for PA and (2) having an adopted child. The inclusion and exclusion criteria in the control group were almost identical to those in the intervention group, except for the recruitment period. Participants in the control group were recruited at 24–27 weeks of gestation.

Before starting the SB reduction program, baseline PA and SB data were collected and the intervention was started at 20 gestational weeks. PA and SB data were also collected at T1 and T2 during the intervention period. Participants completed questionnaires at four time points: baseline, T1, T2, and 1 month after delivery.

The SB reduction program consisted of (1) individual face-to-face guidance, (2) automatic SB alerts using wearable devices, and (3) self-monitoring of SB patterns using the wearable device application.

Individual face-to-face guidance was performed at 20 weeks of gestation by a researcher and five trained midwives using leaflets developed for this program. During a 20-min tutorial that followed the routine prenatal checkup, participants were informed of the American College of Obstetricians and Gynecologists guidelines [3], which recommend PA to maintain health during pregnancy. Participants were informed of the prevalence and adverse effects of prolonged SB among pregnant women. To reduce SB and increase PA, participants were asked to interrupt their sitting position at least once an hour to perform a short activity [23]. The leaflet showed some examples of short activities (e.g., washing of dishes, checking the mailbox, and shredding documents). In addition, participants discussed with the guidance providers, ideas of activities they could do in their own lives, and were asked to fill out a leaflet with these ideas. To confirm the standardization and consistency of the interventions, the researcher received reports from the guidance providers on the participants’ responses and questions.

SB and PA were monitored using the Polar Loop 2 (Polar Electro Oy, Polar, Finland) wearable device [24] between 20 weeks of gestation and delivery. The participants were asked to wear the device on the wrist of their non-dominant arm immediately upon awakening. The device was kept on during the day, except when bathing. The device has an automatic alert feature when SB occurred for more than 1 h. Participants used this automatic alert feature from their initial health guidance session until delivery. Participants were instructed to stand up and engage in a recommended or chosen short activity when notified of an automatic alert, unless they were physically unwell or unable to move freely.

For self-monitoring of SB patterns, participants were asked to use the free application for the wearable device on their mobile devices (Polar Flow mobile app; Polar Electro Oy, Polar, Finland) from the time of providing informed consent. The use of the application and the frequency and content of SB self-monitoring were optional for participants. The application displayed a 24-h pie chart of SB and three levels of PA intensity measured by the device. Participants could see the change in SB and PA during pregnancy and recognize their SB patterns (increased or decreased SB).

Pregnant Japanese women usually receive several health guidance information sessions by midwives during routine prenatal checkups [25]. Midwives also provide information, at around 20 weeks of gestation, about the importance of maintaining PA as a means of weight gain control and preventing gestational diabetes. Midwives also ask about actual daily PA and discuss and set goals of PA levels considering the risks of preterm delivery and the health benefits. When rapid body weight increases are noticed at health checkups, midwives confirm and discuss how to maintain PA levels. In this study, participants in both the intervention and control groups received standard care, including PA guidance, at their healthcare facilities.

PA and SB were assessed using the wearable device at baseline, T1, and T2. Participants were instructed to wear the device during waking hours until delivery and to remove and charge the device at bedtime. The device login account and password were given to the participants by the researcher, who was then able to access and collect data on participants’ PA and SB. 

The device-specific algorithm automatically detects and categorizes daily activities into 5 activity intensity levels per minute: sleep, SB, light PA, moderate PA, and vigorous PA. Data were used when participants wore the device for at least 10 h per day (i.e., the total time of SB, light PA, moderate PA, and vigorous PA was more than 10 h) [26].

Participants’ PA and SB were described as the cumulative time per day spent at each PA intensity level, including SB, light PA, and moderate-to-vigorous PA. The cumulative time of PA or SB within a specific gestational week was defined as the average value from day 0 to day 7 of that week. The cumulative times of PA and SB at T1 and T2 were calculated using the mean values of 4 weeks from 24–27 and 32–35 weeks of gestation, respectively.

Acceptability was assessed based on three self-reported questions at T1. We asked participants the questions: “Did you find the guidance easy to understand?”, “Did you find the guidance useful for you?”, and “Did the guidance match what you thought you had to do?”. Participants were asked to select the most appropriate response: strongly agree, agree, disagree, or strongly disagree.

Feasibility was assessed by the device wearing rate, the response rate to the SB alerts, and the frequency of self-monitoring of the device wearing rate, calculated as the percentage of the number of days worn for more than 10 h per day during the intervention period: 21–35 weeks of gestation (105 days). The response rate to the SB alerts was obtained by self-report at T1 and T2: always (100%), usually (50–80%), sometimes (30–50%), or rarely (less than 30%). The frequency of self-monitoring was self-reported at T1 and T2: twice or thrice a week, once a week, or less than once a week.

Demographic data and pregnancy and delivery parameters (age, weight, exercise habits, pregnancy complications, etc.) were obtained by self-report at baseline, T1, T2, and 1 month after delivery. Participants were asked to answer a questionnaire by accessing a URL provided by email by the researcher at each visit.

Different devices were used to measure PA and SB, in the intervention and control groups. The Polar Loop 2 (Polar Electro Oy, Polar, Finland) [24] was worn by the intervention group, while the Silmee W10 (TDK, Tokyo, Japan) [27] was worn by the control group. To confirm whether the data could be compared between the different devices, PA data were collected from two women who wore both devices simultaneously on their non-dominant wrists for 2 weeks. The correlation coefficients showed a significant positive correlation between the two sets of measurements (Supplementary Figure S1). In the univariate regression model, we found that data from the Silmee W10 were higher than those from the Polar Loop 2 when SB time was less than 500 min, while data from the Silmee W10 were lower than those from the Polar Loop 2 when SB time was more than 500 min. Both light and moderate PA data from the Silmee W10 were lower than those from the Polar Loop 2 regardless of their cumulative times. Therefore, the measurements of the control group were adjusted using a linear regression equation. The effect of SB reduction and increased PA was assessed using analysis of covariance for the difference between the control and intervention groups at T2, with the value at T1 used as a covariate. The mean SB time of pregnant women in the previous study [22] was 521.7 ± 89.5 min, which was approximately 40 min longer than that of Japanese adult women [28]. The sample size of the study was calculated based on the assumption of a mean 40-min decrease in cumulative times of SB in the intervention group compared with the control group. For this effect size with a power of 0.80 at alpha 0.05 (two-sided), we calculated a sample size of 64 participants. Based on an estimated dropout rate of 10%, we aimed to include 70 participants. Descriptive and comparative analyses were performed using IBM SPSS Statistics for Windows, version 24.0 (IBM Corp., Armonk, NY, USA). *p*-Values less than 0.05 were considered to be statistically significant.

## 3. Results

**Figure 1:**
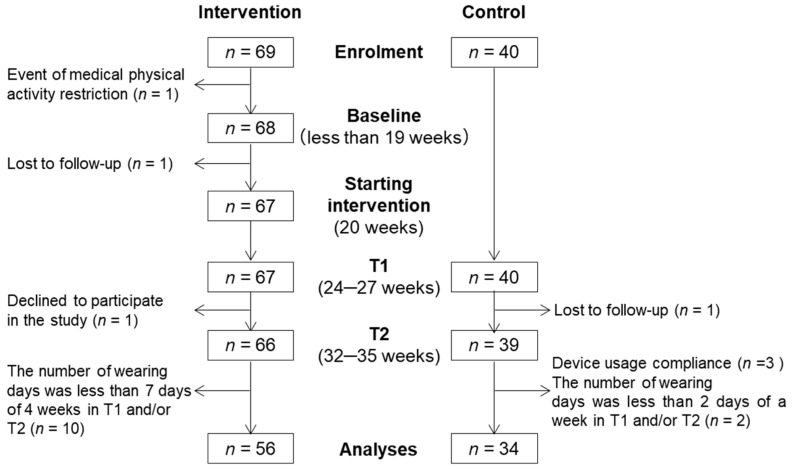
Flowchart of the selection of the study participants.

Of the 69 participants enrolled in the intervention group, 66 (95.7%) completed the program until T2 (Figure 1). There were 10 participants (14.5%) who had less than 7 days of activity data for T1 and T2 and were excluded; thus, the remaining 56 participants (81.2%) were included in the final analysis. Data from the control group at T1 and T2 were obtained from 39 participants. 

The mean age in the intervention and control groups was 33.9 ± 5.5 and 33.6 ± 4.8 years, respectively. Exercise habits and infertility treatment were significantly different between the two groups (*p* = 0.004 and *p* = 0.046, respectively), while there were no significant differences in other characteristics between the intervention and control groups (Table 1).

**Figure 2:**
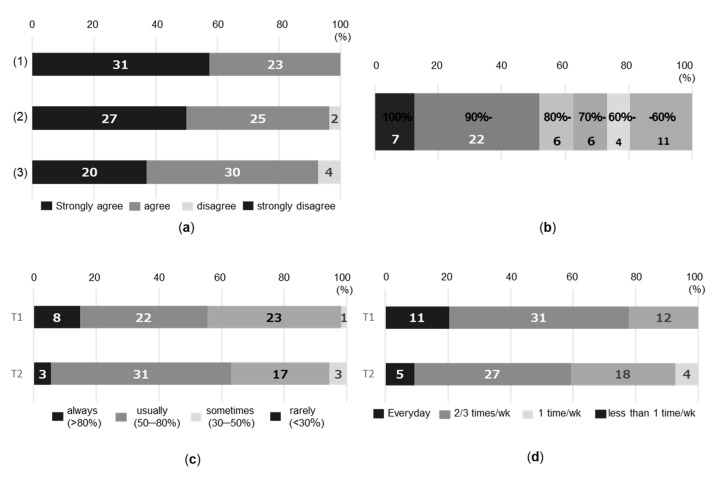
Acceptability and feasibility of the intervention (*n* = 54). (**a**) Acceptability of the health guidance: Answers to the self-reported questions at T1 (Did you find the guidance easy to understand? Did you find the guidance useful for you? Did the guidance match what you thought you had to do?); (**b**) Device wearing rate: the percentage of the number of days the device was worn for more than 10 h per day during the intervention period (21–35 weeks of gestation (105 days)); and (**c**) Response rate to SB alerts: answers to the self-reported questions at T1 and T2; and (**d**) Frequency of self-monitoring: answers to the self-reported questions at T1 and T2. SB, sedentary behavior.

Regarding the three questions about the acceptability of the intervention, more than 90% of participants answered strongly agree or agree (Figure 2a). The mean number of days the device was worn for more than 10 h per day at 21–35 weeks of gestation (105 days) was 90.2 days. Twenty-nine participants (52%) fully wore the device for more than 90% of the days measured, while 11 participants (20%) reported a full wearing rate of less than 60% (Figure 2b). The response to automatic SB alerts was reported as always or usually by 30 participants (55.5%) at T1 and 34 participants (63.0%) at T2 (Figure 2c). Regarding the frequency of self-monitoring, the number of participants who reported every day decreased from 11 participants (20.4%) at T1 to five participants (9.3%) at T2, and participants who reported twice or thrice a week decreased from 31 participants (57.4%) at T1 to 27 participants (50.0%) at T2 (Figure 2d).

**Figure 3:**
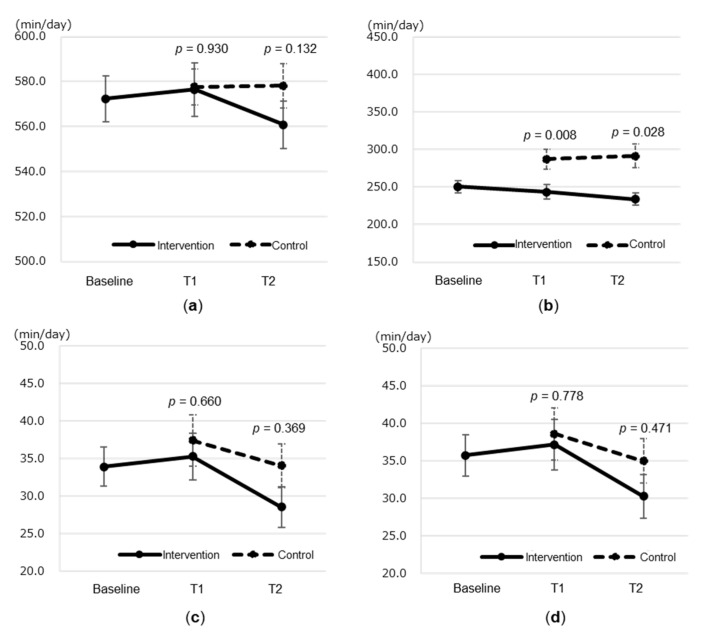
Effects of sedentary behavior (SB) and physical activity (PA) in the intervention and control groups. (**a**) SB, (**b**) Light PA, (**c**) Moderate PA, and (**d**) Moderate-to vigorous PA. Plots of the mean values at three time points. Solid lines represent the intervention group, and dotted lines represent the control group. Error bars indicate the mean error. T1: Student’s *t*-test was used to compare the two groups. T2: Analysis of covariance was used to compare the two groups (fixed effects: intervention/control; covariates: values at T1). Intervention group: baseline (*n* = 56), T1 (*n* = 55), T2 (*n* = 54). Control group: T1/T2 (*n* = 34).

The cumulative time of light PA at T1 was lower in the intervention group than in the control group (243.4 ± 72.0 vs. 287.4 ± 77.1 min; *p* < 0.001), while there were no significant differences in other PA levels or SB at T1 between the two groups (Figure 3). There was no significant difference in the cumulative time of SB at T2 between the two groups, even using the T1 value as a covariate (560.9 ± 77.1 vs. 578.1 ± 57.6 min; F = 2.31; *p* = 0.132), while the cumulative time of light PA at T2 was significantly different between the two groups (234.0 ± 61.1 vs. 291.6 ± 92.4 min; F = 5.01; *p* = 0.028).

Compared with the control group, the gestational weeks at delivery in the intervention group were significantly earlier (39.3 ± 1.2 vs. 40.0 ± 1.0 weeks; *t* = −2.92; *p* = 0.004) and the birth weight significantly lower (3026.2 ± 377.2 vs. 3189.2 ± 312.1 g; *t* = −2.04; *p* = 0.04), while there were no significant differences in other delivery parameters between the two groups (Table 2).

## 4. Discussion

In this study, we conducted an SB reduction program for pregnant women that included individual face-to-face guidance, automatic alerts during SB from wearable devices, and self-monitoring. This study showed that this program is acceptable and feasible for pregnant women. However, this study reported a low response rate to automatic SB alerts and a low frequency of self-monitoring, and it was unclear whether this intervention reduced SB or not.

Disease prevention strategies during pregnancy have major public health implications for both mother and child. Despite the benefits of PA during pregnancy for maternal and child health [1,2], many pregnant women remain inactive [5,6,7]. It is difficult to engage pregnant women in PA because of the barriers to exercise [15,16,17]. The findings of this study are significant in that they show that our approach of interrupting SB is acceptable and feasible for pregnant women, despite no significant reduction in SB.

Most participants completed the intervention program and wore the device almost every day during the intervention period, and the individual face-to-face guidance was acceptable. This result is consistent with two previous studies that reported high adherence in populations in whom it is traditionally difficult to promote exercise, such as obese adults or the elderly [20,21]. In addition, the use of a smartphone application and wearable device may have resulted in high feasibility. Choi et al. [29] conducted a PA intervention program using mobile technologies, which resulted in high acceptability and feasibility among pregnant women. It appears that an SB reduction program using wearable devices can also be applied to pregnant women.

Despite our hypothesis, there was no difference in SB reduction from T1 to T2 between the intervention and control groups, even when the T1 value was used as a covariate. One possible reason for this could have been that the participants interrupted their SB, but the duration of interruption might have been shorter than expected. It is possible that the participants became accustomed to the self-monitoring and alerts, thus ignoring them during the long intervention period and causing the cumulative SB time to not decrease.

The absence of a difference may be due to differences in exercise habits during pregnancy between the two groups; no one continued to exercise in the intervention group, whereas 17.6% of participants continued to exercise in the control group. This suggests that the control group was not averse to being active; hence, they were able to maintain a higher baseline PA level and lower gestation PA reduction. Therefore, this could explain why no difference in SB reduction was detected. Another possible reason may be seasonal changes in PA. Previous studies reported that PA increases in the summer season, compared with the winter season [30,31]. The intervention group was surveyed throughout the year, and few seasonal changes in PA were found (data not shown), while the control group was surveyed during the summer season. These factors may explain the higher PA levels in the control group. Further studies are warranted in which these factors are controlled, to determine the effectiveness of SB reduction.

Because the researcher could access the participants’ PA and SB data at any time, it is possible that the participants felt observed by the researcher, which may have prompted behavioral change. However, this effect is common to both the intervention and control groups; therefore, it would not have affected the results.

The response rate to automatic SB alerts was low, which suggests that participants did not take action to interrupt SB when notified, even though they were advised on PA during the guidance sessions. It would be difficult to recall the details of PA performed and take further action when participants have been focusing on daily tasks, such as workplace meetings. Cooley et al. [23] conducted a workplace computer-based intervention program in which participants were required to select one activity from examples on a computer screen when the computer remained on for more than 1 h, resulting in increased interruption of SB. Adding such an approach to this program to encourage participants to take action toward PA would be essential to increase PA during pregnancy, by improving response rates to automatic SB alerts.

The frequency of self-monitoring decreased from T1 to T2. Our program included instructions on self-monitoring in the individual health guidance sessions, but the contents were left to the participants. Therefore, it would be difficult for participants to select appropriate changes in behavior based on self-monitoring SB and PA. Self-monitoring is a behavior change technique that contributes the most to intervention effectiveness in any health behavior [32] and provides feedback, leading to more effective self-monitoring and increased self-efficacy [33]. This program would need to include the following elements concerning changes in PA and SB for a few weeks: feedback, encouragement, reinforcement, and motivational messages.

Comparisons of pregnancy and delivery parameters showed a significant difference in the gestational age at delivery and birth weight of the babies; however, all of them were normal-term babies with a birth weight appropriate for gestational age. These were not clinically significant differences; hence, this program was not associated with an increase in adverse events.

This study has several limitations. First, the devices worn by the intervention and control groups were different, because the device worn by the control group was not available for the intervention group. The measurement latency of the two devices was adjusted; however, potential errors could still occur, which may have affected the results. Second, we did not recruit participants for the intervention and control groups simultaneously, resulting in differences in the demographic data between the two groups, which might have affected the results. Finally, the sample size was set to evaluate the acceptability and feasibility of the program. This study could not statistically evaluate the effectiveness of the program. We showed good acceptability and feasibility of the program. Further research with sufficient sample sizes is needed to evaluate the effectiveness of the program in reducing SB and preventing maternal complications.

## 5. Conclusions

The SB reduction program was found to be acceptable and feasible for pregnant women; however, the SB reduction effect of the intervention remains unclear. This program needs to be improved to increase the response rate to automatic SB alerts and the frequency of self-monitoring. In future studies, it will be necessary to improve the intervention and evaluate the effectiveness of the program in randomized controlled trials.

## Figures and Tables

**Table 1:** Characteristics of the study participants.

Characteristic ^1^	Intervention (*n* = 56)		Control (*n* = 34)		*p*-Value
Age (years)	34.4 ± 5.1		33.6 ± 4.8		0.453
≤35	31	(55.4)	21	(61.8)	0.551
>35	25	(44.6)	13	(38.2)	–
Pre-pregnancy BMI (kg/m^2^)	21.6 ± 4.0		21.6 ± 2.9		0.945
≤24.5	49	(87.5)	31	(91.2)	0.737
>24.5	7	(12.5)	3	(8.8)	–
Infertility treatment					
No	33	(58.9)	27	(79.4)	0.046
Yes	23	(41.1)	7	(20.6)	–
Past history					
No	34	(60.7)	25	(73.5)	0.215
Yes	22	(39.3)	9	(26.5)	–
Thyroid disease	8	–	2	–	–
Gynecological disease	5	–	0	–	–
Hyperlipidemia	2	–	0	–	–
Atopic dermatitis	1	–	6	–	–
Other	6	–	1	–	–
Employment					
Maternity leave/No	17	(30.4)	11	(32.4)	0.843
Yes	39	(69.6)	23	(67.6)	–
Exercise habit					
No	32	(57.1)	18	(52.9)	0.004
Stopped	24	(42.9)	10	(29.4)	–
Yes	0	(0.0)	6	(17.6)	–

^1^ Mean ± standard deviation or *n* (%), χ^2^ test or Fisher’s exact test. BMI, body mass index.

**Table 2:** Pregnancy and delivery parameters.

Parameter ^1^	Intervention (*n* = 47)		Control (*n* = 33)		*t*-Value	*p*-Value
Mode of delivery						
Vaginal delivery	37	(78.7)	25	(75.8)	–	0.754
Cesarean section	10	(21.3)	8	(24.2)	–	–
Breech position	4	–	1	–	–	–
Prior uterine surgery	4	–	0	–	–	–
Anomaly of rotation	2	–	2	–	–	–
Non-reassuring fetal status	0	–	1	–	–	–
Unknown	0	–	4	–	–	–
Hospitalization of an infant						
No	44	(93.6)	32	(37.0)	–	0.639
Yes	3	(6.4)	1	(3.0)	–	–
Neonatal respiratory disorder	3	–	0	–	–	–
Physiologic hyperbilirubinemia	1	–	0	–	–	–
Pneumothorax	0	–	1	–	–	–
Gestational age at delivery (weeks)	39.3 ± 1.2		40.0 ± 1.0		−2.92	0.004
Birth weight (g)	3026.2 ± 377.2		3189.2 ± 312.1		−2.04	0.045
Total labor time (minutes)	649.7 ± 431.8		818.2 ± 619.0		−1.26	0.211
Intrapartum blood loss (mL)	594.6 ± 293.4		759.7 ± 549.0		−1.46	0.153

^1^ Mean ± standard deviation or *n* (%), χ^2^ test, Fisher’s exact test, or Student’s *t*-test.

## Supplementary Materials

The following is available online at https://www.mdpi.com/2227-9032/8/4/439/s1, Figure S1: Scatter plots and linear regression equations for the simultaneous measurements from Silmee W10 and Polar Loop 2: (a) SB, (b) light PA, (c) moderate PA, and (d) vigorous PA. PA, physical activity; SB, sedentary behavior.
