# Supplementary material for: Acceptability and Feasibility of a Sedentary Behavior Reduction Program during Pregnancy: A Semi-Experimental Study
Source: Healthcare (Basel). 2020 Oct 29;8(4):439. doi: 10.3390/healthcare8040439 (PMC7712505; doi:10.3390/healthcare8040439)
Supplement: Supplementary file 1 [file healthcare-08-00439-s001.pdf]

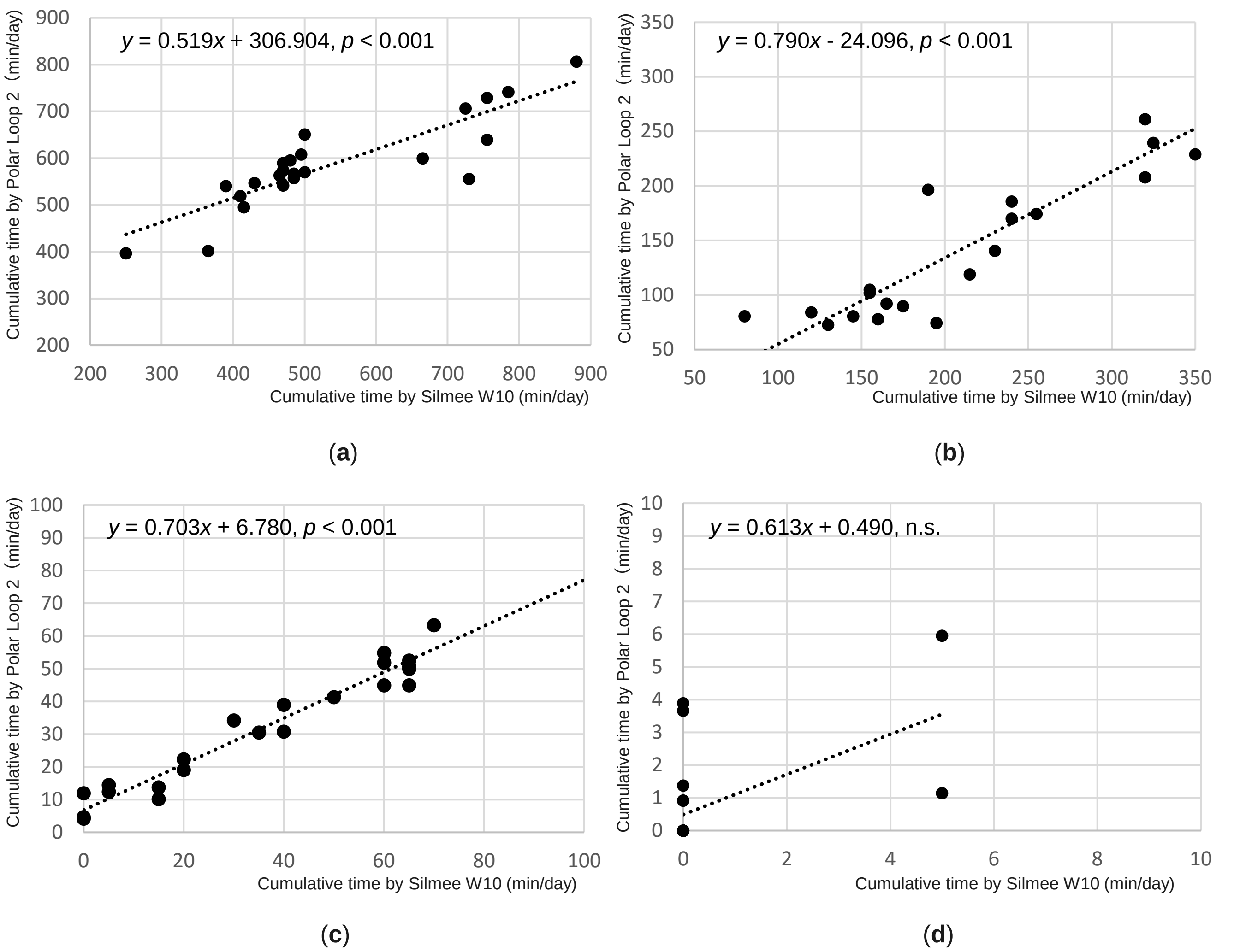

Figure S1: Scatter plots and linear regression equations for the simultaneous measurements from Silmee W10 and Polar Loop 2 : (**a**) SB, (**b**) light PA, (**c**) moderate PA, and (**d**) vigorous PA. PA, physical activity; SB, sedentary behavior.
